# The Walled Lake

**Published:** 1890-01

**Authors:** 


### THE WALLED LAKE.

One of the wonders of the United States, is the Walled Lake, in Iowa. It covers a surface of 2,800 acres, with a depth of twenty-five feet of water. It is from two to three feet higher than the surrounding country, and is enclosed by a wall ten feet high, fifteen feet wide at the bottom, and sloping up to five feet wide at the top. The stones of which the wall is built, vary in size from one weighing one hundred pounds, to those weighing three tons. Around the entire lake is a belt of trees, half a mile in width; with this exception, the country in which the lake is located, is a rolling prairie. When, how, or by whom, this wall was constructed, or these trees set out, is a mystery.

HAIR FALLING OFF.—This is undoubtedly becoming much commoner, and setting in much earlier in life. In plain English, there is no certain remedy for it. So many jottings and recipes have been published on the subject that one is quite at a loss respecting them. When there are fifty infallible remedies for a common ailment, the probability is that they all more often disappoint than give satisfaction. Wash the head with a good soap every other day, and rub in a spirit hair-wash or cantharides wash in the evening, and a little glycerine and borax in the morning. Do this for a month.—PHYSICIAN.

